# The amino acid transporter LAT1 coordinates proper motor function at the perinatal stage

**DOI:** 10.1038/s41419-026-08663-8

**Published:** 2026-03-24

**Authors:** Koki Sadamori, Manami Hiraiwa, Tetsuhiro Horie, Kazuya Tokumura, Kazuya Fukasawa, Kentaro Sahashi, Soji Hayashida, Takuya Kubo, Makoto Yoshimoto, Shohei Tsuji, Yasuhito Ishigaki, Masahisa Katsuno, Eiichi Hinoi

**Affiliations:** 1 Department of Bioactive Molecules, Pharmacology, Gifu Pharmaceutical University, Gifu, Japan; 2 Medical Research Institute, Kanazawa Medical University, Kahoku, Japan; 3 Department of Pharmacy, Kanazawa Medical University Hospital, Kahoku, Japan; 4 Department of Neurology, Nagoya University Graduate School of Medicine, Nagoya, Japan; 5 United Graduate School of Drug Discovery and Medical Information Sciences, Gifu University, Gifu, Japan; 6 Center for One Medicine Innovative Translational Research (COMIT), Division of Innovative Modality Development, Institute for Advanced Study, Gifu University, Gifu, Japan

**Keywords:** Motor neuron, Motor neuron disease

## Abstract

L-type amino acid transporter 1 (LAT1, encoded by *Slc7a5*) contributes to amino acid homeostasis and signaling in numerous cell types. Several lines of evidence implicate LAT1 in mammalian central nervous system development, but its functional significance in specific neuronal subtypes is largely unknown. Here, we demonstrate that LAT1/*Slc7a5* expression in synapsin 1 (Syn1)-expressing neurons is essential for motor circuit development and motor coordination at the perinatal stage. Mice lacking *Slc7a5* in Syn1-expressing neurons exhibited progressive motor coordination deficits and early postnatal lethality. These deficits were associated with selective degeneration of lower spinal motor neurons, reactive gliosis, skeletal muscle atrophy, and maldevelopment of neuromuscular junctions (NMJs), but no abnormalities in gross brain structure or neuronal viability. Pharmacological inhibition of apoptosis prolonged the survival of *Slc7a5-*deficient mice and reduced both lower motor neuron loss and NMJ maldevelopment. Furthermore, multi-cohort transcriptome analyses revealed inactivation of amino acid transport activity along with the downregulation of *Slc7a5* expression in motor neurons of spinal muscular atrophy model mice. These results suggest that the amino acid transport system is essential for the survival and function of lower spinal motor neurons during early postnatal development, and identifies LAT1 as a potential therapeutic target for early-onset motor neuron diseases.

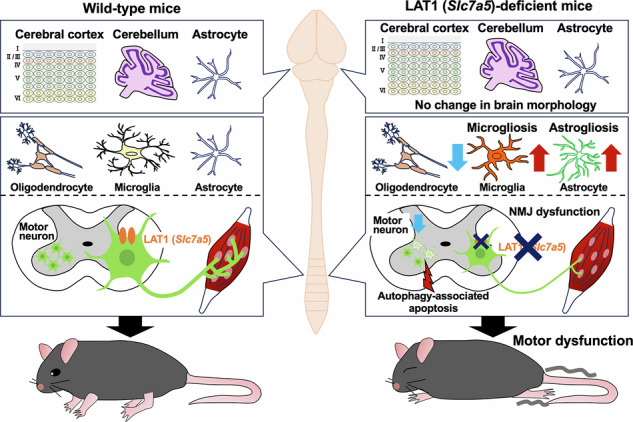

## Introduction

L-type amino acid transporter 1 (LAT1) facilitates the cellular uptake of large neutral amino acids, including branched-chain amino acids (BCAAs) such as leucine, isoleucine, and valine via exchange with intracellular amino acids [1–3]. Unlike specialized transporters for charged amino acids, LAT1 functions independently of the transmembrane Na^+^ and pH gradients. LAT1 is essential for amino acid sensing and signaling in specific cell types, thereby regulating myriad cellular processes, including gene expression, the synthesis of proteins and other macromolecules, energy metabolism, and various anabolic pathways [4–6]. Expression is also upregulated in various cancer cell types, where LAT1 contributes to hyperactive proliferation. Thus, overexpression is frequently associated with accelerated tumor growth and poorer prognosis, suggesting that LAT1 is a promising target for diagnostic cancer imaging and anticancer therapeutics [7, 8].

The expression of LAT1/*Slc7a5* is limited to specific tissues under physiological conditions, but may be upregulated more broadly in response to processes requiring tissue growth and remodeling [9, 10]. For instance, we recently demonstrated a pivotal role for LAT1/*Slc7a5* in bone remodeling and cartilage homeostasis through its cell-specific inactivation in osteoclasts and chondrocytes, respectively [11, 12]. Global *Slc7a5* deficiency was also reported to induce neurodevelopment defects and embryonic lethality in mice [10, 13]. In addition, *Slc7a5* ablation in blood–brain barrier (BBB) cells, especially endothelial cells, induced severe neurological abnormalities such as motor dysfunction and an autism spectrum disorder (ASD)-like phenotype in mice, whereas *Slc7a5* deletion in forebrain excitatory neurons altered neuronal excitability and resulted in progressive microcephaly [14, 15]. Pathogenic mutations in the encoding gene *SLC7A5* are also associated with a wide range of neuropathological features in humans, such as microcephaly, ASD, and motor coordination problems [14, 16, 17]. We recently reported that genetic inactivation of *Slc7a5* in leptin receptor-expressing hypothalamic neurons led to metabolic and skeletal abnormalities [18]. Further, we have shown that neuronal *Slc7a5* expression is induced by hypoxia, a pathogenic state common to numerous otherwise distinct neurodegenerative diseases [19].

These previous reports showing expression and critical functions of LAT1/*Slc7a5* in distinct neuronal populations suggest broader roles in central nervous system (CNS) development, homeostasis, and pathophysiology. A more comprehensive understanding of these functions requires systematic examination in different neuronal populations. Accordingly, in the current study, we examined LAT1 functions in synapsin 1 (Syn1)-expressing neurons of conditional knockout mice generated by crossing *Slc7a5*-floxed mice with *Syn1-Cre* transgenic mice [20]. Deletion of LAT1/*Slc7a5* in Syn1-expressing neurons resulted in a progressive motor impairment phenotype characterized by loss of coordination during the early perinatal period accompanied by selective degeneration of lower spinal motor neurons, skeletal muscle atrophy, maldevelopment of neuromuscular junctions (NMJs), and early lethality, but without gross abnormalities in upper motor neurons or CNS structure. Finally, both *Slc7a5* expression and amino acid transporter activity were impaired in motor neurons of spinal muscular atrophy (SMA) model mice. These findings suggest that deficient neutral amino acid transport system in the perinatal period may contribute to the pathogenesis of early-onset motor neuron diseases.

## Results

### *Slc7a5* deletion in Syn1-expressing neurons progressively impaired motor function test performance during the perinatal stage

**Fig. 1:**
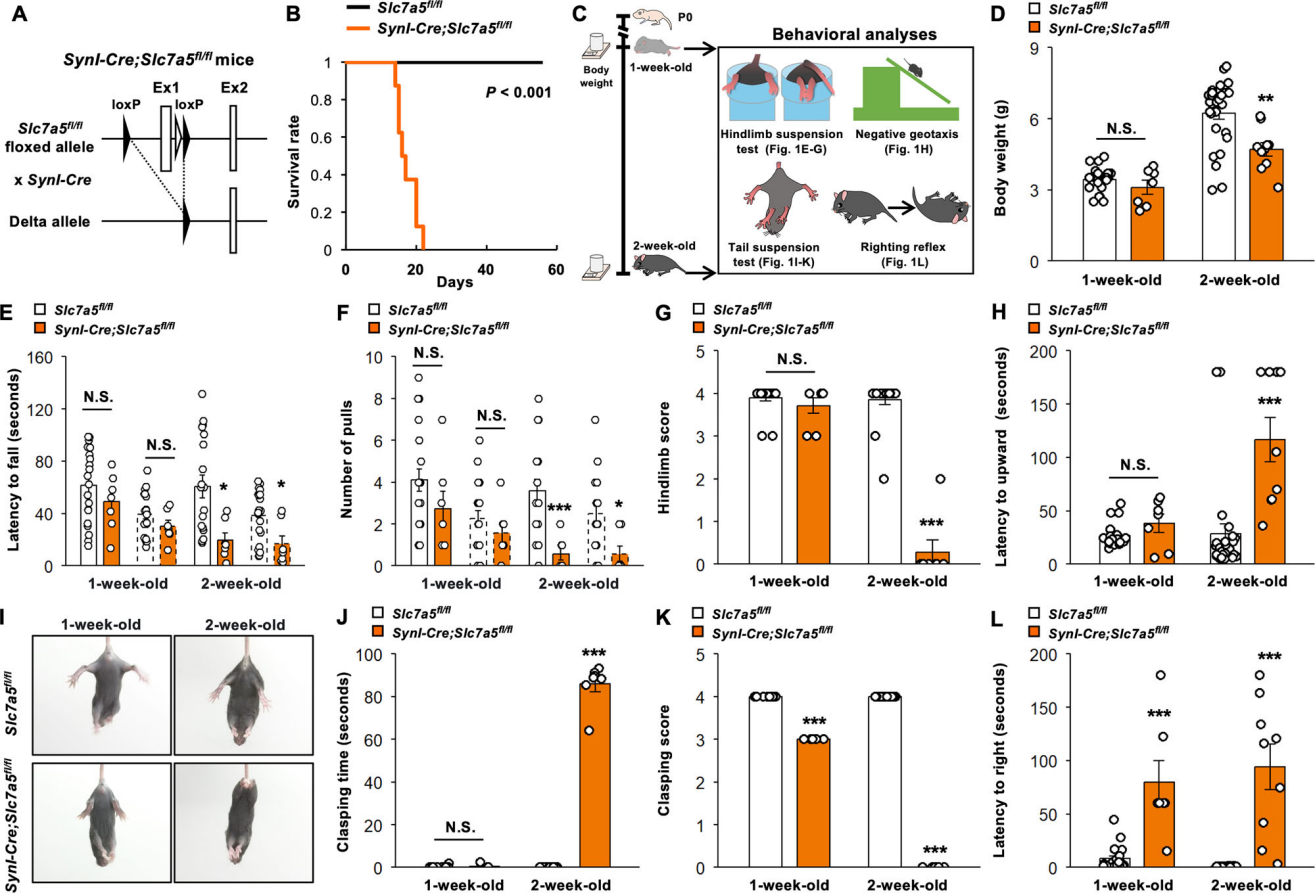
*Slc7a5* ablation in Syn1-expressing neurons causes motor dysfunction at perinatal stage. **A** Schematic diagram of generation of neuron-specific *Slc7a5* knockout mice. **B** Kaplan–Meier survival analysis of *Slc7a5*^*fl/fl*^ (*n* = 8) and *Syn1-Cre;Slc7a5*^*fl/fl*^ mice (*n* = 12). **C** Schematic diagram of behavioral analyses in *Slc7a5*^*fl/fl*^ and *Syn1-Cre;Slc7a5*^*fl/fl*^ mice. **D** Body weight of *Slc7a5*^*fl/fl*^ (1 week: *n* = 20; 2 week: *n* = 22) and *Syn1-Cre;Slc7a5*^*fl/fl*^ mice (1 week: *n* = 7; 2 week: *n* = 10). **E**–**G** Quantitative data of hindlimb suspension test; (**E**) latency to fall (seconds), (**F**) number of pulls, and (**G**) hindlimb score of *Slc7a5*^*fl/fl*^ (1 week and 2 week: *n* = 20) and *Syn1-Cre;Slc7a5*^*fl/fl*^ mice (1 week and 2 week: *n* = 7). Solid bars represent the results of the first trial, while dashed bars represent the results of the second trial. **H** Quantitative data of negative geotaxis test; latency to upward (seconds) of *Slc7a5*^*fl/fl*^ (1 week: *n* = 20; 2 week: *n* = 25) and *Syn1-Cre;Slc7a5*^*fl/fl*^ mice (1 week: *n* = 7; 2 week: *n* = 9). **I**–**K** Representative pictures and quantitative data of tail suspension test (self-clasping test); (**J**) clasping time (seconds), and (**K**) clasping score of *Slc7a5*^*fl/fl*^ (1 week: *n* = 11; 2 week: *n* = 21) and *Syn1-Cre;Slc7a5*^*fl/fl*^ mice (1 week: *n* = 7; 2 week: *n* = 8). **L** Quantitative data of righting reflex test; latency to right (seconds) of *Slc7a5*^*fl/fl*^ (1 week: *n* = 20; 2 week: *n* = 25) and *Syn1-Cre;Slc7a5*^*fl/fl*^ mice (1 week: *n* = 7; 2 week: *n* = 9). NS not significant. *P < 0.05, **P < 0.01, and ***P < 0.001.

To evaluate the physiological functions of LAT1 in Syn1-expressing neurons during CNS development, we bred Syn1 neuron-specific *Slc7a5* knockout mice by crossing *Slc7a5*-floxed mice with *Syn1-Cre* transgenic mice (Fig. 1A). Mice lacking *Slc7a5* in Syn1-expressing cells, hereafter referred to as *Syn1-Cre;Slc7a5*^*fl/fl*^ mice, were born at the frequency predicted by Mendelian principles and appeared physically normal at birth (data not shown). However, these *Syn1-Cre;Slc7a5*^*fl/fl*^ mice rapidly developed poor motor skills and abnormal gait as early as one week of age, and none survived beyond 21 days of age (Fig. 1B). To further assess the extent of their motor deficits, we conducted a series of behavioral tests (Fig. 1C). While body weight distribution was similar to control littermates at one week of age, *Syn1-Cre;Slc7a5*^*fl/fl*^ mice were significantly underweight by two weeks of age (Fig. 1D). In the hindlimb suspension test, latency to fall, number of pulls, and hindlimb scores did not differ between genotypes at one week of age. At two weeks of age, however, *Syn1-Cre;Slc7a5*^*fl/fl*^ mice demonstrated a significantly shorter latency to fall, fewer pulls, and a lower hindlimb score than control mice, indicating progressive onset of hindlimb muscle weakness, fatigue, and general neuromuscular dysfunction (Fig. 1E–G). Further, *Syn1-Cre;Slc7a5*^*fl/fl*^ mice displayed a significant increase in latency to upward movement during the negative geotaxis test at two weeks of age but not at one week of age, indicating progressively impaired motor coordination or balance (Fig. 1H). These mice also exhibited a significant increase in hindlimb clasping time at two weeks of age and a significant decrease in clasping score at both one and two weeks of age during tail suspension, consistent with progressive motor coordination deficits (Fig. 1I–K). Finally, *Syn1-Cre;Slc7a5*^*fl/fl*^ mice demonstrated a significantly longer latency to righting during the surface righting reflex test at both one and two weeks of age (Fig. 1L). Collectively, these results suggest that *Slc7a5* deficiency in Syn1 neurons results in a constellation of behavioral phenotypes suggestive of motor neuron dysfunction.

### *Slc7a5* deficiency in neurons leads to the degeneration of motor neurons in the lumbar spinal cord

**Fig. 2:**
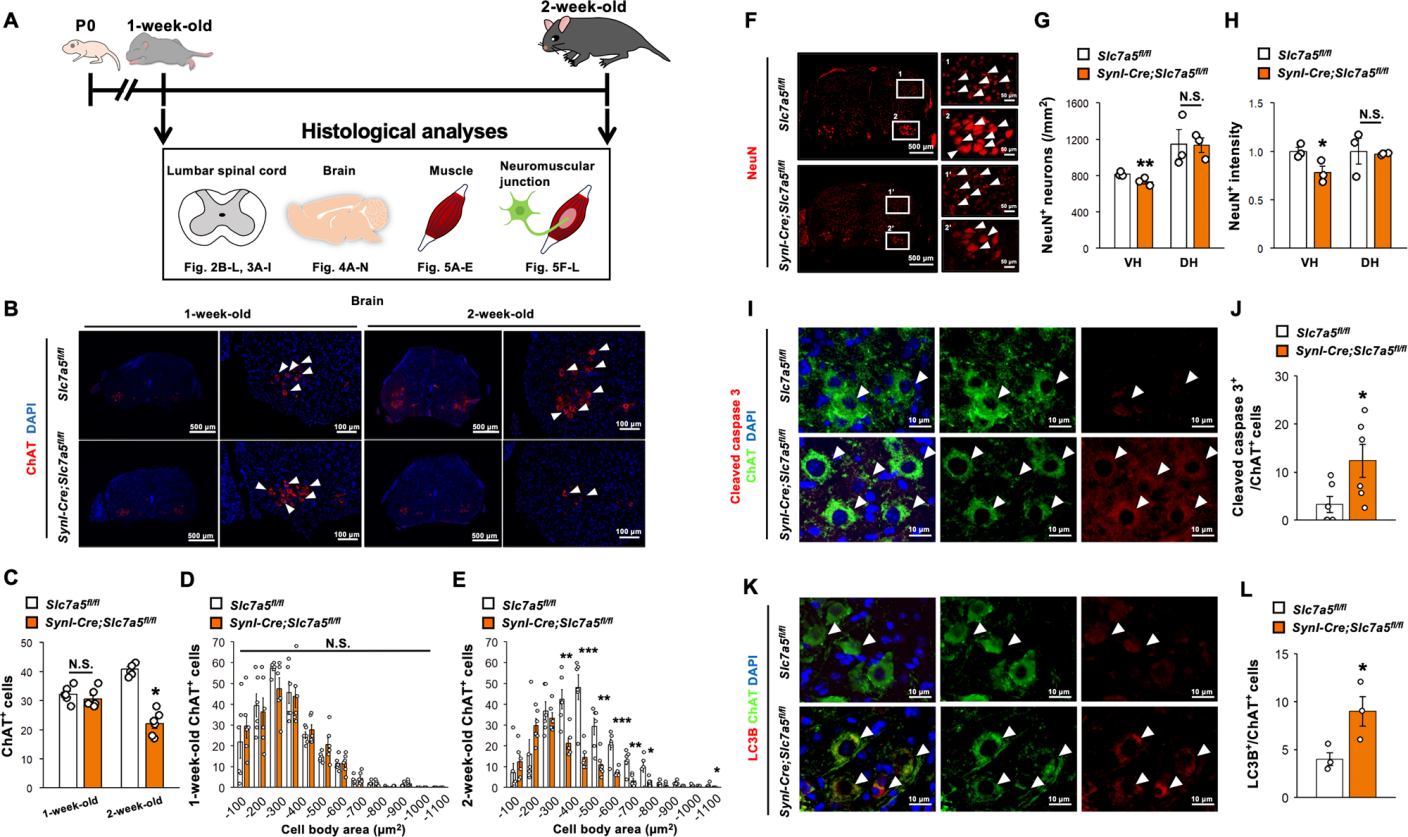
*Slc7a5* ablation in Syn1-expressing neurons leads to motor neurons degeneration in lumbar spinal cord. **A** Schematic diagram of histological analyses in *Slc7a5*^*fl/fl*^ and *Syn1-Cre;Slc7a5*^*fl/fl*^ mice. **B**–**E** Immunofluorescent images and quantitative data of ChAT/DAPI staining in lumbar spinal cord; (**C**) the number of ChAT positive cell, and (**D**, **E**) the distribution of cell body area (μm^2^) of *Slc7a5*^*fl/fl*^ (1 week and 2 week: *n* = 6) and *Syn1-Cre;Slc7a5*^*fl/fl*^ mice (1 week and 2 week: *n* = 6). Arrowheads indicate representative ChAT positive cells. **F**–**H** Immunofluorescent images and quantitative data of NeuN staining in lumbar spinal cord; (**G**) the number of NeuN positive cell (/mm^2^), and (**H**) NeuN immunointensity of *Slc7a5*^*fl/fl*^ (*n* = 3) and *Syn1-Cre;Slc7a5*^*fl/fl*^ mice (*n* = 3) at 2 weeks of age. Arrowheads indicate representative NeuN-positive cells. **I**, **J** Immunofluorescent images and quantitative data of cleaved caspase 3/ChAT/DAPI staining in lumbar spinal cord; (**J**) the number of cleaved caspase 3 and ChAT double positive cells of *Slc7a5*^*fl/fl*^ (*n* = 6) and *Syn1-Cre;Slc7a5*^*fl/fl*^ mice (*n* = 6) at 1 week of age. Arrowheads indicate representative cleaved caspase 3, ChAT, and double-positive cells. **K**, **L** Immunofluorescent images and quantitative data of LC3B/ChAT/DAPI staining in lumbar spinal cord; (**L**) the number of LC3B and ChAT double positive cells of *Slc7a5*^*fl/fl*^ (*n* = 3) and *Syn1-Cre;Slc7a5*^*fl/fl*^ mice (*n* = 3) at 1 week of age. Arrowheads indicate representative LC3B, ChAT, and double positive cells. NS not significant, VH ventral horn, DH dorsal horn. *P < 0.05, **P < 0.01, and ***P < 0.001.

We then histologically examined whether impaired motor function in neuron-specific *Slc7a5*-deficient mice was associated with regional or widespread neuronal damage and dysfunction (Fig. 2A). Both α- and γ-motor neurons can be identified by immunostaining for choline acetyltransferase (ChAT) and distinguished by higher NeuN expression in α-motor neurons relative to γ-motor neurons [21]. The number of ChAT-positive motor neurons was reduced by approximately 50% in the lumbar spine ventral horn of *Syn1-Cre;Slc7a5*^*fl/fl*^ mice at two weeks of age compared with control littermates (Fig. 2B, C). In particular, the number of larger ChAT-positive motor neurons (more than 400 μm^2^ in cross-sectional area) was significantly reduced in *Syn1-Cre;Slc7a5*^*fl/fl*^ mice at two weeks of age (Fig. 2E). On the contrary, there were no significant differences in the number, distribution, and size of ChAT-positive motor neurons between *Syn1-Cre;Slc7a5*^*fl/fl*^ and control mice at one week of age (Fig. 2B–D). Both the number of large NeuN-positive motor neurons and NeuN staining intensity were reduced in the lumbar spine ventral horn of *Syn1-Cre;Slc7a5*^*fl/fl*^ mice at two weeks of age compared with control littermates, suggesting region-specific loss of α-motor neurons (Fig. 2F–H). Further, double immunostaining for ChAT and the apoptosis marker cleaved caspase-3 revealed fourfold more frequent apoptosis in the ventral horn of *Syn1-Cre;Slc7a5*^*fl/fl*^ mice than control littermates at one week of age (prior to the onset of motor neuron loss) (Fig. 2I, J). Double immunostaining for ChAT and the autophagosome marker light chain 3B (LC3B) also revealed an increase in autophagosome number within ChAT-positive motor neurons of *Syn1-Cre;Slc7a5*^*fl/fl*^ mice compared with control littermates (Fig. 2K, L). Together, these data indicate that *Slc7a5* deficiency in Syn1-positive neurons led to the early loss of ventral horn α-motor neurons, at least in part due to greater autophagy-associated apoptosis.

### *Slc7a5* deficiency in neurons leads to gliosis and myelopathy in the lumbar spinal cord

**Fig. 3:**
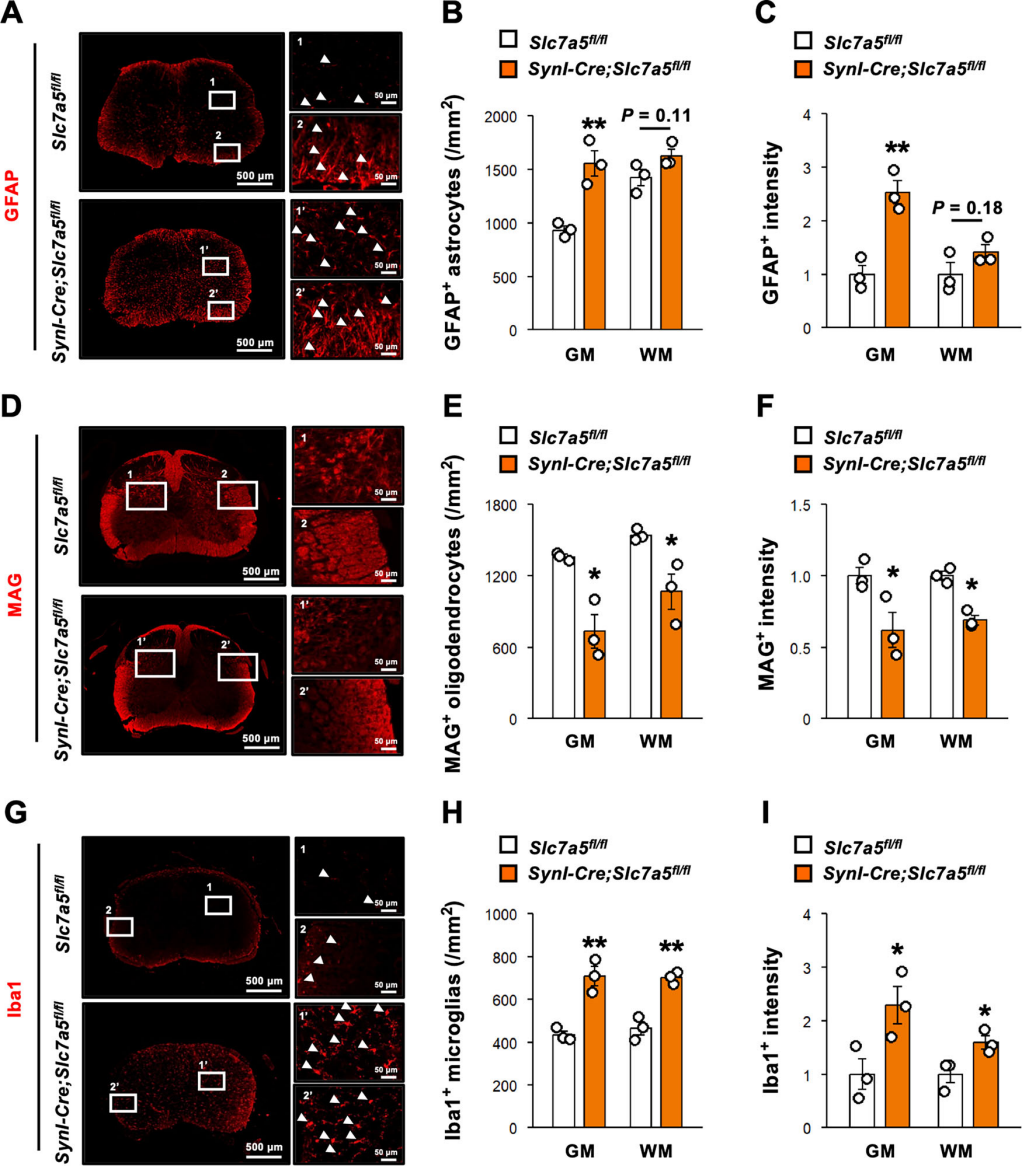
*Slc7a5* ablation in Syn1-expressing neuron results in gliosis and myelopathy in lumbar spinal cord. **A**–**C** Immunofluorescent images and quantitative data of GFAP staining in lumbar spinal cord; (**B**) the number of GFAP positive cell (/mm^2^), and (**C**) GFAP immunointensity of *Slc7a5*^*fl/fl*^ (*n* = 3) and *Syn1-Cre;Slc7a5*^*fl/fl*^ mice (*n* = 3) at 2 weeks of age. Arrowheads indicate representative GFAP-positive cells. **D**–**F** Immunofluorescent images and quantitative data of MAG staining in lumbar spinal cord; (**E**) the number of MAG positive cell (/mm^2^), and (**F**) MAG immunointensity of *Slc7a5*^*fl/fl*^ (*n* = 3) and *Syn1-Cre;Slc7a5*^*fl/fl*^ mice (*n* = 3) at 2 weeks of age. **G**–**I** Immunofluorescent images and quantitative data of Iba1 staining in lumbar spinal cord; (**H**) the number of Iba1 positive cell (/mm^2^), and **(I)** Iba1 immunointensity of *Slc7a5*^*fl/fl*^ (*n* = 3) and *Syn1-Cre;Slc7a5*^*fl/fl*^ mice (*n* = 3) at 2 weeks of age. Arrowheads indicate representative Iba1 positive cells. NS not significant, GM gray matter, WH white matter. *P < 0.05 and **P < 0.01.

Neurodegeneration in the brain and spinal cord is usually associated with reparative and pathological glial cell responses collectively termed reactive transformation [22], so we conducted immunostaining for markers of these responses as an adjunct to motor function testing and neuronal histochemistry. Immunostaining revealed greater numbers of GFAP-positive astrocytes and greater GFAP staining intensity in the gray matter but not the white matter of *Syn1-Cre;Slc7a5*^*fl/fl*^ mice at two weeks of age compared with control littermates, a sign of reactive astrogliosis in response to local neuronal damage (Fig. 3A–C). Immunostaining for the transmembrane myelin-associated glycoprotein (MAG) also revealed a reduced number of MAG-expressing cells and decreased expression intensity in both gray and white matter of *Syn1-Cre;Slc7a5*^*fl/fl*^ mice at two weeks of age, suggesting loss of myelin-producing oligodendrocytes (Fig. 3D–F). Finally, immunostaining revealed significantly greater numbers of cells expressing the ionized calcium-binding adapter molecule 1 (Iba1), a marker of ramified, activated, ameboid, and dystrophic microglia, as well as greater expression intensity in both the gray and white matter of *Syn1-Cre;Slc7a5*^*fl/fl*^ mice at two weeks of age (Fig. 3G–I). Collectively, these findings indicate that *Slc7a5* deficiency in neurons triggers astrogliosis, microgliosis, and myelopathy in the lumbar spinal cord.

### *Slc7a5* deficiency in neurons does not alter brain morphology

**Fig. 4:**
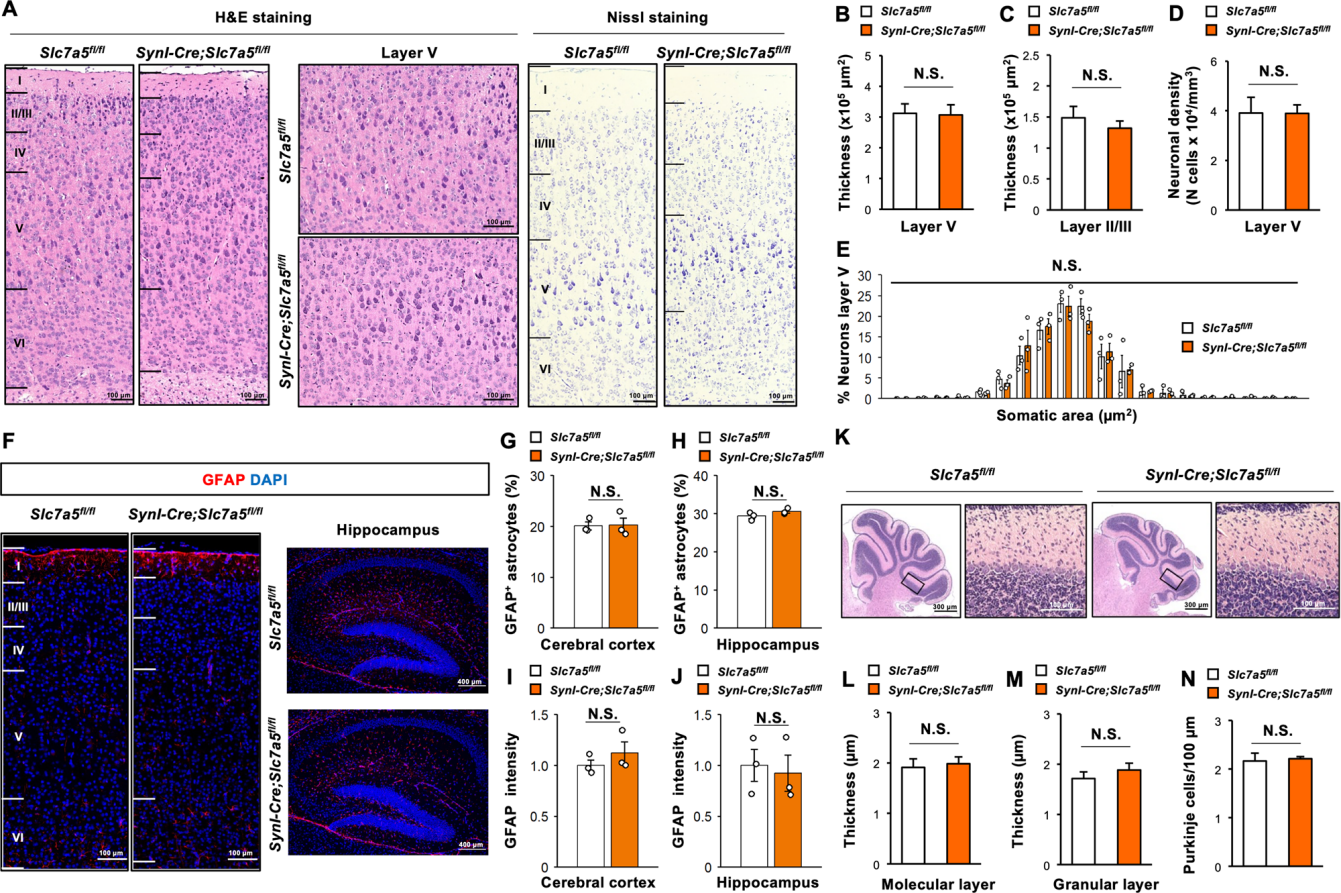
*Slc7a5* ablation in Syn1-expressing neuron does not induce abnormalities of brain morphology. **A**–**E** H&E and Nissl staining images and quantitative data of the area of (**B**) Layer V and (**C**) Layer II/III of cerebral cortex (×10^5^ μm^2^), (**D**) the neuronal density in Layer V of cerebral cortex (N cells ×10^4^/mm^3^), and (**E**) the distribution of somatic area in Layer V of cerebral cortex (μm^2^) of *Slc7a5*^*fl/fl*^ (*n* = 3) and *Syn1-Cre;Slc7a5*^*fl/fl*^ mice (*n* = 3) at 2 weeks of age. **F**–**J** Immunofluorescent images and quantitative data of GFAP/DAPI staining in cerebral cortex and hippocampus; the % of GFAP positive cells in (**G**) cerebral cortex and (**H**) hippocampus, and the GFAP immunointensity in (**I**) cerebral cortex and (**J**) hippocampus of *Slc7a5*^*fl/fl*^ (*n* = 3) and *Syn1-Cre;Slc7a5*^*fl/fl*^ mice (*n* = 3) at 2 weeks of age. **K-N**. H&E staining images and quantitative data of the thickness of **(L)** molecular layer and **(M)** granular layer of cerebellum (μm), and **(N)** the number of Purkinje cells in cerebellum (/mm) of *Slc7a5*^*fl/fl*^ (*n* = 3) and *Syn1-Cre;Slc7a5*^*fl/fl*^ mice (*n* = 3) at 2 weeks of age. NS not significant.

The observed deficits in motor strength and coordination could also reflect neuronal damage or dysfunction in the motor cortex and cerebellum [23], so we conducted further histological analyses of *Syn1-Cre;Slc7a5*^*fl/fl*^ mouse brain. General cortical morphology and motor cortex thickness (layers I to VI) appeared normal in both *Syn1-Cre;Slc7a5*^*fl/fl*^ mice and control littermates at two weeks of age according to hematoxylin−eosin (H&E) and Nissl staining (Fig. 4A). In addition, motor cortex layer V and layer II/III thicknesses were comparable between *Syn1-Cre;Slc7a5*^*fl/fl*^ mice and control littermates (Fig. 4B, C), and both layer V neuronal density and size distribution were indistinguishable between genotypes (Fig. 4D, E). Moreover, no apparent gliosis was observed in the cerebral cortex and hippocampus of *Syn1-Cre;Slc7a5*^*fl/fl*^ mice at two weeks of age as neither the number of GFAP-positive astrocytes nor GFAP staining intensity differed significantly from control littermates (Fig. 4G–J). In addition, gross cerebellar morphology appeared normal in *Syn1-Cre;Slc7a5*^*fl/fl*^ mice at two weeks of age according to H&E staining (Fig. 4K). The thicknesses of the cerebellar molecular and granular layers and the number of Purkinje cells also did not differ significantly between *Syn1-Cre;Slc7a5*^*fl/fl*^ mice and control littermates (Fig. 4L–N). Therefore, *Slc7a5* deficiency did not lead to gross morphological abnormalities or region-specific brain damage that could account for the motor coordination deficits in *Syn1-Cre;Slc7a5*^*fl/fl*^ mice.

### *Slc7a5* deficiency in neurons leads to muscle atrophy and NMJ maldevelopment

**Fig. 5:**
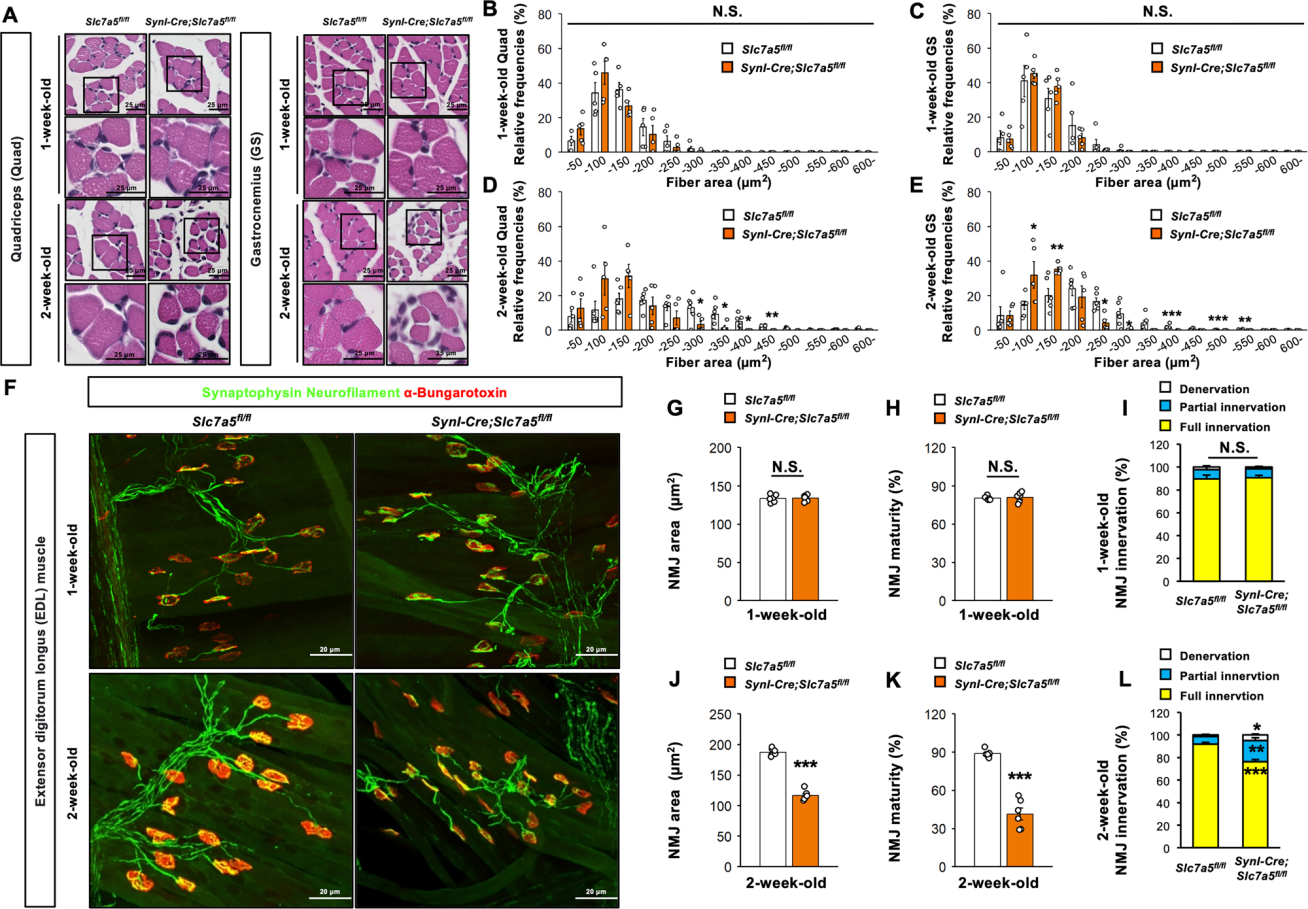
*Slc7a5* ablation in Syn1-expressing neuron leads to muscle atrophy and NMJ dysfunction. **A**–**E** H&E staining images and quantitative data of the distribution of fiber area (μm^2^) of (**B**, **D**) quadriceps and (**C**, **E**) gastrocnemius of *Slc7a5*^*fl/fl*^ (1 week and 2 week: *n* = 5, 6) and *Syn1-Cre;Slc7a5*^*fl/fl*^ mice (1 week and 2 week: *n* = 5). **F**–**L** Immunofluorescent images of synaptophysin, neurofilament, and BTX staining and quantitative data of NMJ in EDL muscle; (**G**, **J**) NMJ area (μm^2^), (**H**, **K**) NMJ maturity (%), and (**I**, **L**) NMJ innervation (denervation, partial innervation, and full innervation) (%) of *Slc7a5*^*fl/fl*^ (1 week and 2 week: *n* = 6) and *Syn1-Cre;Slc7a5*^*fl/fl*^ mice (1 week and 2 week: *n* = 6). NS not significant. *P < 0.05, **P < 0.01, and ***P < 0.001.

The general motor weakness and weight loss observed in *Slc7a5*-deficient mice also suggested that degeneration of lower motor neurons resulted in muscular atrophy and maldevelopment of NMJs. Histological analyses of skeletal muscles isolated from one-week-old *Syn1-Cre;Slc7a5*^*fl/fl*^ mice (prior to overt motor impairment) revealed no apparent morphological abnormalities and no significant differences in the cross-sectional area distributions of quadriceps and gastrocnemius myofibers compared with control littermates (Fig. 5A–C). In contrast, at two weeks of age, the quadriceps and gastrocnemius of *Syn1-Cre;Slc7a5*^*fl/fl*^ mice exhibited fewer larger myofibers, a sign of progressive muscle atrophy (Figs. 5A, D, E).

We then examined possible changes in NMJ structure and skeletal muscle innervation density by dual staining for postsynaptic acetylcholine receptors (AChRs) with α-bungarotoxin (BTX) and presynaptic synaptophysin and neurofilaments using targeted antibodies. These staining methods revealed no significant genotype difference in NMJ structure, area, maturity, and innervation within the extensor digitorum longus (EDL) muscle of *Syn1-Cre;Slc7a5*^*fl/fl*^ mice compared with control littermates at one week of age (Fig. 5F–H). However, *Syn1-Cre;Slc7a5*^*fl/fl*^ mice exhibited abnormalities in NMJ structure, area, maturity, and innervation within the EDL muscle at two weeks of age compared with control littermates (Fig. 5F, J, and K). To quantify these changes, motor endplates were classified as “fully innervated,” “partially innervated,” or “denervated” according to the staining patterns of AChRs and nerve terminals, and the proportions compared between genotypes. While these proportions did not differ between genotypes at one week of age, the proportions of denervated and only partial innervated endplates were significantly greater while the proportion classified as fully innervated was significantly reduced in *Syn1-Cre;Slc7a5*^*fl/fl*^ mice compared with control mice at two weeks of age (Fig. 5I, L). Collectively, these results show that *Slc7a5* deficiency results in progressive muscular atrophy as well as NMJ maldevelopment, manifested as reduced topological complexity.

### Pharmacological inhibition of apoptosis prolongs survival, ameliorates motor neuron loss, and reduces MNJ maldevelopment in *Slc7a5*-deficient mice

**Fig. 6:**
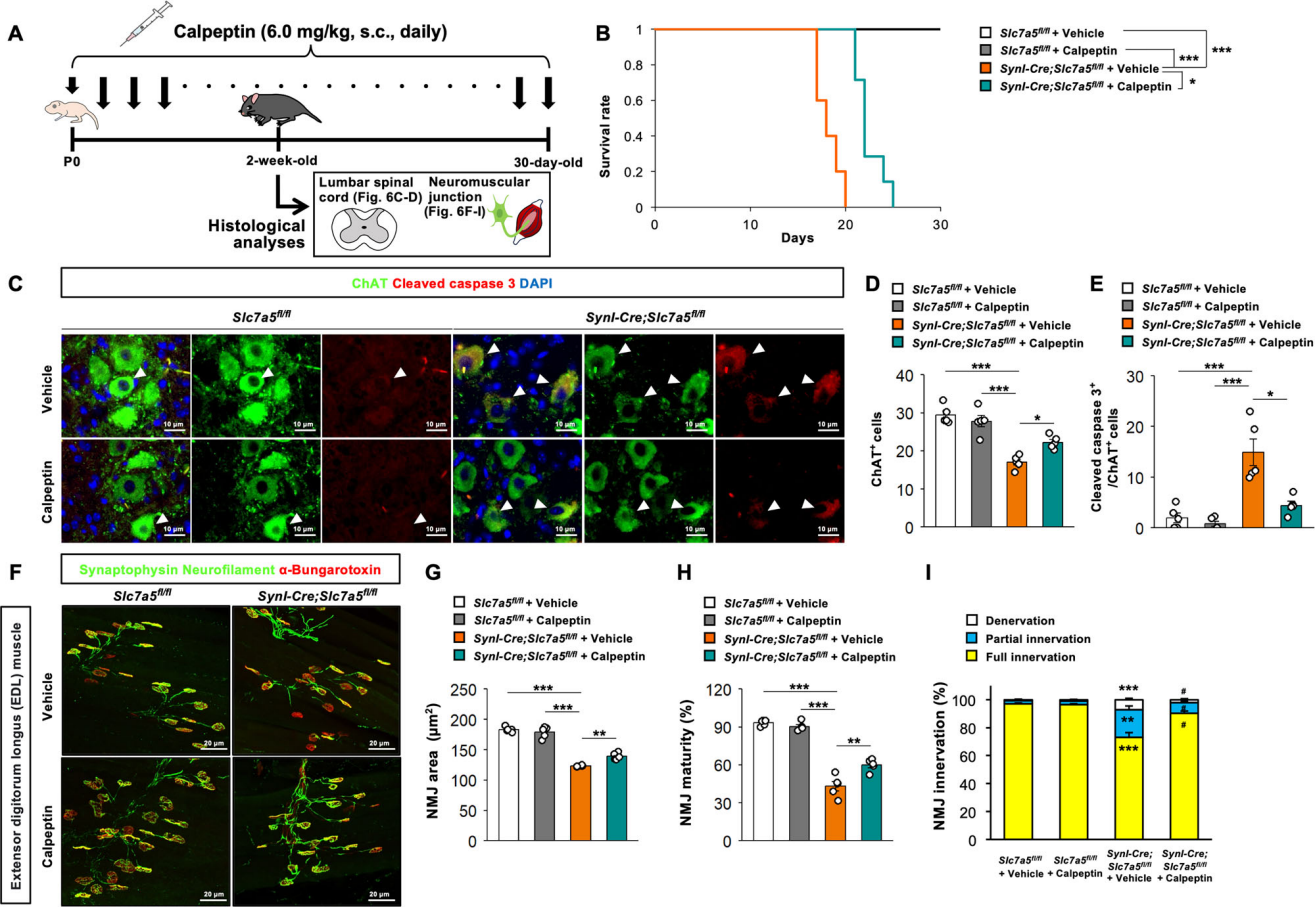
Inhibition of apoptosis ameliorates the phenotypes induced by *Slc7a5* deficiency. **A** Schematic diagram of calpeptin administration schedule followed by histological analyses in *Slc7a5*^*fl/fl*^ and *Syn1-Cre;Slc7a5*^*fl/fl*^ mice. **B** Kaplan–Meier survival analysis of *Slc7a5*^*fl/fl*^ mice and *Syn1-Cre;Slc7a5*^*fl/fl*^ mice treated with vehicle or calpeptin (*Slc7a5*^*fl/fl*^ mice + vehicle, *n* = 9; *Slc7a5*^*fl/fl*^ mice + calpeptin, *n* = 7; *Syn1-Cre;Slc7a5*^*fl/fl*^ mice + vehicle, *n* = 5; *Syn1-Cre;Slc7a5*^*fl/fl*^ mice + calpeptin, *n* = 7). **C**–**E** Immunofluorescent images and quantitative data of cleaved caspase 3/ChAT/DAPI staining in lumbar spinal cord; (**D**) the number of ChAT positive cells, and (**E**) the number of cleaved caspase 3 and ChAT double positive cells of *Slc7a5*^*fl/fl*^ mice and *Syn1-Cre;Slc7a5*^*fl/fl*^ mice treated with vehicle or calpeptin (*Slc7a5*^*fl/fl*^ mice + vehicle, *n* = 5; *Slc7a5*^*fl/fl*^ mice + calpeptin, *n* = 5; *Syn1-Cre;Slc7a5*^*fl/fl*^ mice + vehicle, *n* = 5; *Syn1-Cre;Slc7a5*^*fl/fl*^ mice + calpeptin, *n* = 5). Arrowheads indicate representative cleaved caspase 3, ChAT, and double positive cells. **F**–**I** Immunofluorescent images of synaptophysin/neurofilament/BTX staining and quantitative data of NMJ in EDL muscle; (**G**) NMJ area (μm^2^), (**H**) NMJ maturity (%), and (**I**) NMJ innervation (%) of *Slc7a5*^*fl/fl*^ mice and *Syn1-Cre;Slc7a5*^*fl/fl*^ mice treated with vehicle or calpeptin (*Slc7a5*^*fl/fl*^ mice + vehicle, *n* = 5; *Slc7a5*^*fl/fl*^ mice + calpeptin, *n* = 5; *Syn1-Cre;Slc7a5*^*fl/fl*^ mice + vehicle, *n* = 5; *Syn1-Cre;Slc7a5*^*fl/fl*^ mice + calpeptin, *n* = 5). *P < 0.05, **P < 0.01, ***P < 0.001, and ^#^*P* < 0.05.

To examine if the observed increase in spinal motor neuron apoptosis rate contributes to motor neuron loss and impaired NMJ development in *Syn1-Cre;Slc7a5*^*fl/fl*^ mice, we examine if pharmacologic apoptosis inhibition could rescue these pathological phenotypes (Fig. 6A). Indeed, daily administration of calpeptin, a potent cell-penetrating calpain inhibitor demonstrated to attenuate apoptosis [24], significantly prolonged the survival of *Syn1-Cre;Slc7a5*^*fl/fl*^ mice but had no effect on the survival of control mice (Fig. 6B). Moreover, calpeptin administration significantly increased the total number of ChAT-positive motor neurons while reducing the number of apoptotic ChAT-positive motor neurons in the lumbar ventral horn of *Syn1-Cre;Slc7a5*^*fl/fl*^ mice but not control littermates (Fig. 6C–E). Finally, calpeptin administration significantly increased NMJ area and maturity within the EDL muscle of *Syn1-Cre;Slc7a5*^*fl/fl*^ mice but had no influence on NMJ integrity in control mice (Fig. 6F–H). The proportions of denervated and partially innervated myofibers were significantly reduced in EDL muscle of calpeptin-treated *Syn1-Cre;Slc7a5*^*fl/fl*^ mice, while the proportion of fully innervated endplates was significantly higher compared to vehicle-treated *Syn1-Cre;Slc7a5*^*fl/fl*^ mice (Fig. 6I). Taken together, these results indicate that apoptosis contributes to the observed motor neuron degeneration and NMJ maldevelopment in *Slc7a5*-deficient mice.

### Association with SMA model mice

**Fig. 7:**
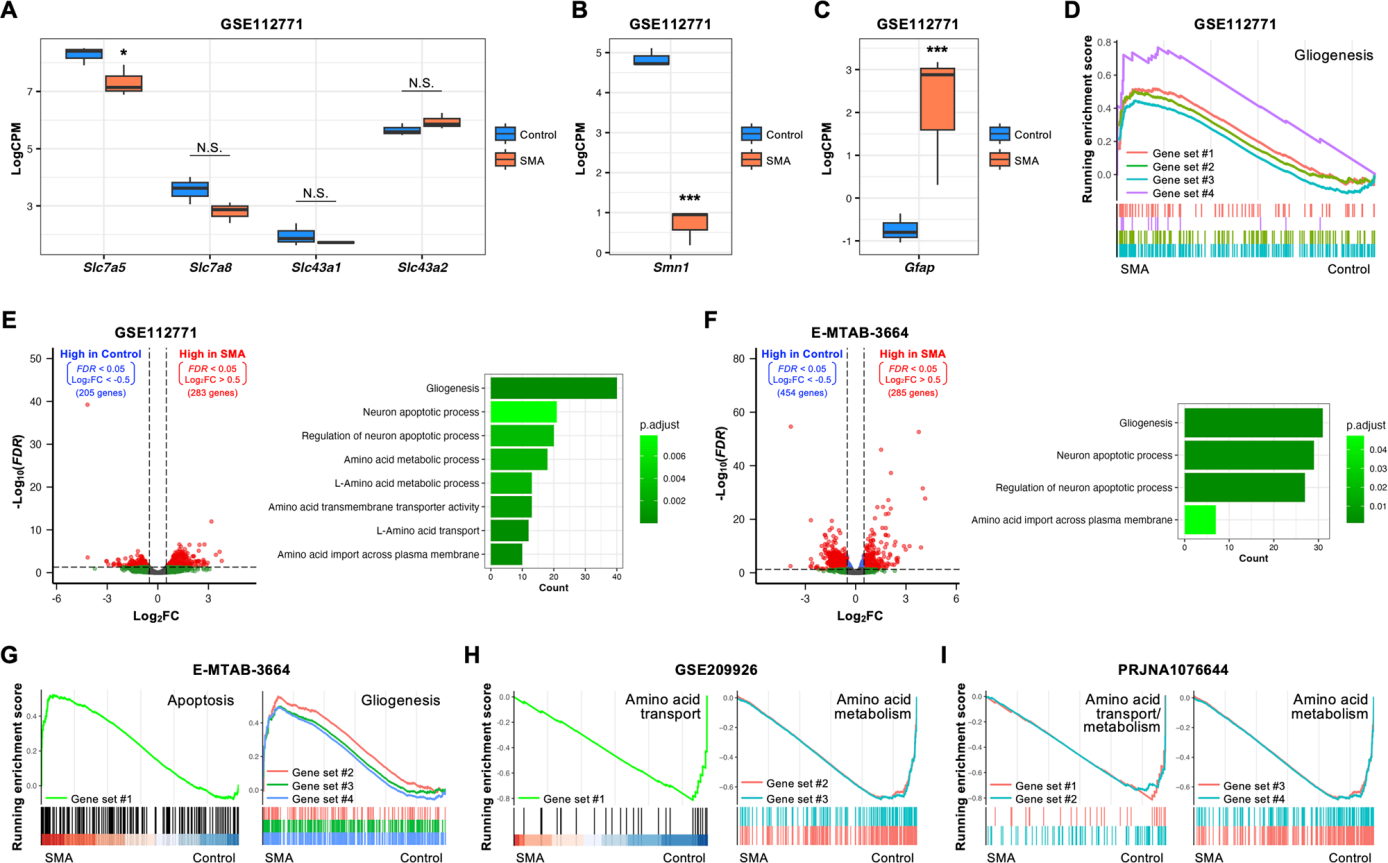
Bioinformatic analyses suggest the involvement of amino acid signaling in SMA model mice. The expression of (**A)** *Slc7a5* (LAT1), *Slc7a8* (LAT2), *Slc43a1* (LAT3), *Slc43a2* (LAT4), (**B**) *Smn1*, and (**C**) *Gfap* in the GSE112771 dataset (Control, *n* = 3; SMA, *n* = 3). **D** The result of GSEA related to gliogenesis in the GSE112771 dataset. Gene set #1, GOBP_ASTROCYTE_DIFFERENTIATION (NES = 1.92, *P*_adj_ = 0.005); Gene set #2, GOBP_GLIAL_CELL_DEVELOPMENT (NES = 2.01, *P*_adj_ < 0.001); Gene set #3, GOBP_GLIAL_CELL_DIFFERENTIATION (NES = 1.96, *P*_adj_ < 0.001); Gene set #4, GOBP_GLIAL_CELL_FATE_COMMITMENT (NES = 2.06, *P*_adj_ = 0.015). **E** Volcano plot (left) and the result of GO analysis (right) in the GSE112771 dataset. **F** Volcano plot (left) and the result of GO analysis (right) in the E-MTAB-3664 dataset. **G** The result of GSEA related to apoptosis (left) and gliogenesis (right) in the E-MTAB-3664 dataset. Gene set #1, HALLMARK_APOPTOSIS (NES = 1.61, *P*_adj_ = 0.023); Gene set #2, GOBP_GLIAL_CELL_DEVELOPMENT (NES = 1.72, *P*_adj_ = 0.049); Gene set #3, GOBP_GLIAL_CELL_DIFFERENTIATION (NES = 1.63, *P*_adj_ < 0.001); Gene set #4, GOBP_GLIOGENESIS (NES = 1.64, *P*_adj_ = 0.006). **H** The result of GSEA related to amino acid transport (left) and amino acid metabolism (right) in the GSE209926 dataset. Gene set #1, REACTOME_AMINO_ACID_TRANSPORT_ACROSS_THE_PLASMA_MEMBRANE (NES = −1.66, *P*_adj_ = 0.011); Gene set #2, GOBP_AMINO_ACID_METABOLIC_PROCESS (NES = −1.49, *P*_adj_ < 0.001); Gene set #3, GOBP_L_AMINO_ACID_METABOLIC_PROCESS (NES = −1.49, *P*_adj_ = 0.001). **I** The result of GSEA related to amino acid transport (left) and amino acid metabolism (right) in the PRJNA1076644 dataset. Gene set #1, REACTOME_AMINO_ACID_TRANSPORT_ACROSS_THE_PLASMA_MEMBRANE (NES = −1.66, *P*_adj_ = 0.007); Gene set #2, WP_AMINO_ACID_METABOLISM (NES = −1.62, *P*_adj_ < 0.001); Gene set #3, GOBP_AMINO_ACID_METABOLIC_PROCESS (NES = −1.50, *P*_adj_ < 0.001); Gene set #4, GOBP_L_AMINO_ACID_METABOLIC_*P*ROCESS (NES = −1.50, *P*_adj_ = 0.002). NES normalized enrichment score, NS not significant. ^*^P < 0.05 and ^***^P < 0.001.

The LAT family, which consists of LAT1 (*Slc7a5*), LAT2 (*Slc7a8*), LAT3 (*Slc43a1*), and LAT4 (*Slc43a2*), transports neutral amino acids into cells and may have redundant functions. Analysis of a bulk RNA-seq dataset of SMA model mice (GSE112771) [25] revealed that *Slc7a5* expression was the highest among the LAT family in motor neurons isolated from control mice (*Smn*^*+/+*^; *SMN2* mice), and its expression was significantly downregulated in motor neurons isolated from SMA model mice (*Smn*^*−/−*^; *SMN2* mice), along with downregulation of *Smn1* expression and increased glial cell properties (Fig. 7A–D). Moreover, gene ontology (GO) analysis of GSE112771 revealed that differentially expressed genes (DEGs) between control and SMA model mice were related to amino acid transport and metabolic activities, as well as gliogenesis and neuron apoptosis (Fig. 7E). Consistently, GO analysis of a different cohort (E-MTAB-3664) [26] showed that DEGs between control mice (*Smn*^*+/−*^; *SMN2*^*tg/0*^ mice) and SMA model mice (*Smn*^*−/−*^; *SMN2*^*tg/0*^ mice) were associated with amino acid transport activity, alongside enrichment of glial cell properties and neuron apoptosis in SMA model mice (Fig. 7F, G). Furthermore, gene set enrichment analysis (GSEA) of the bulk RNA-seq dataset (GSE209926) [27] and the whole-transcriptome sequencing dataset (WTS/RNA-seq) (PRJNA1076644) [28] revealed that amino acid transport and metabolic activities were negatively enriched in SMA model mice (*Smn*^*−/−*^;*SMN2*^*tg/0*^ mice) (Fig. 7H, I). On the contrary, *SLC7A5* expression in spinal cords was comparable between control and ALS patients (Supplementary Fig. 1A–F). Collectively, bioinformatics analyses across different cohorts of SMA model mice suggest inactivation of amino acid transport activity along with downregulation of *Slc7a5* expression in motor neurons of SMA model mice.

## Discussion

Patients with an *SLC7A5* mutation exhibit severe motor deficits [14]. In contrast, *Slc7a5* ablation by *Emx1-Cre* mice, which mediate recombination in the neurons of the neocortex and hippocampus, as well as in the glial cells of the pallium, displays moderate motor deficits [15]. In this study, we used *Syn1-Cre* transgenic mice, which are generally considered to have pan-neuronal recombination activity [20]. *Syn1* is widely expressed in the nervous system, and the *Syn1* promoter drives widespread neuronal expression in adult mice. However, *Syn1-Cre* recombinase activity is largely restricted to the brainstem and spinal cord during the early postnatal period [29]. This restriction may contribute to the progressive and early-onset disease with severe motor deficits and early postnatal lethality caused by *Slc7a5* inactivation in Syn1-positive neurons, although we cannot exclude the possibility that lower motor neurons are highly and uniquely dependent on LAT1 activity during the perinatal stage. In the context of the significance of selective vulnerability in neurodegenerative diseases, further investigation is warranted into the extent to which the introduction of *Slc7a5*/LAT1 in motor neurons within the spinal cord of *Syn1-Cre;Slc7a5*^*fl/fl*^ mice could lead to the rescue of the observed phenotypes (e.g., motor neuron loss, NMJ defects, and survival).

Motor neuron diseases such as amyotrophic lateral sclerosis (ALS) and SMA are characterized by earlier lower motor neuron degeneration, leading to limb muscle wasting and atrophy as initial impairments [30, 31]. While ALS is primarily an adult-onset disorder ultimately destroying both lower and upper motor neurons, SMA primarily afflicts children and is characterized by loss of lower motor neurons along with NMJ breakdown [32, 33]. The *Syn1-Cre;Slc7a5*^*fl/fl*^ mice examined in the current study appeared normal at birth and exhibited the expected Mendelian frequency distribution, indicating the absence of genotype-dependent embryonic lethality. However, they developed rapid motor deficits associated with motor neuron degeneration and impaired NMJ development at the perinatal stage, showing a phenotypic resemblance to mouse models of SMA. Concomitantly, our in silico study of motor neurons in SMA model mice revealed reduced *Slc7a5* expression and impaired amino acid transporter activity. It would be interesting to see whether restoring *Slc7a5*/LAT1 can rescue SMA phenotypes in cellular and animal models. Collectively, these findings support the hypothesis of a functional link between the amino acid transport system and SMA pathophysiology.

Autophagy is a physiological recycling process that degrades long-lived proteins and organelles in lysosomes for the reuse of component elements [34, 35]. Neurons are very long-lived and so are highly dependent on autophagy to replace damaged proteins and organelles [36]. However, autophagy can both protect neurons from potential injury and contribute to injury under different conditions [37]. Accumulation of autophagic vesicles has been reported in the postmortem brains of patients with neurodegenerative disorders and in various mouse models of neurodegeneration, suggesting that maladaptive autophagy contributes to neurodegeneration and disease progression. Indeed, inhibition of autophagy was found to delay motor neuron degeneration and extend the lifespan of SMA model mice [38]. Autophagy is also closely associated with apoptosis, and both processes may be controlled by common or closely interacting signaling pathways [39]. Consistent with a close association, autophagy rate was enhanced in ventral horn motor neurons of *Syn1-Cre;Slc7a5*^*fl/fl*^ mice. Therefore, it is of interest to examine if inhibition of autophagy can also rescue the abnormalities of *Slc7a5*-deficient mice.

The transition from embryonic to postnatal life is the most complex adaptative phase during the entire lifespan [40]. The transition from maternal–fetal exchange via blood vessels to ingestion via the gastrointestinal tract involves shifts in whole-body metabolic homeostasis including dynamic metabolic changes in neurons [41]. Thus, disruption of this adaptation at the perinatal stage may detrimentally influence neurodevelopmental processes and induce neurodevelopmental disorders [42]. Neurons in the cerebral cortex show enhanced reliance on BCAAs as substrates for ATP production during the perinatal period, and this phase is associated with LAT1 upregulation [15, 41]. Recent studies indicate that timely and specific interventions during critical periods can reorient abnormal developmental trajectories in animal models of neurological and neuropsychiatric disorders [43, 44]. Our study further underscores the importance of a specific perinatal time window for neurological motor deficits associated with abnormal amino acid transport system. The current findings highlight the critical role of the amino acid transporter LAT1 in motor neuron homeostasis and suggest a novel molecular link between amino acid sensor in the CNS and motor neuron disorders. We suggest that manipulation of LAT1/*Slc7a5* in lower motor neurons represents a plausible diagnostic and therapeutic strategy for protecting against motor neuron disorders in humans.

## Materials and methods

### Mice

*Syn1-Cre* mice (#003966) were obtained from Jackson Laboratory. *Slc7a5*^*fl/fl*^ mice were crossed with *Syn1-Cre* mice. These mutant mice were backcrossed more than five generations with C57BL/6 J mice. Genotyping was performed by PCR using tail genomic DNA. Mice were bred under standard animal housing conditions at 23°C ± 1°C with a relative humidity of 55% and a light/dark cycle of 12 h, with free access to food and water. The study protocol meets the guidelines of the Japanese Pharmacological Society and was approved by the Committee for the Ethical Use of Experimental Animals at Gifu Pharmaceutical University (2024-011R1) and Gifu University (AG-P-N-20250163). The number of animals used per experiment is stated in the figure legends. Genomic DNA was extracted, and subsequent PCR with specific primers to validate the deletion efficiency as described previously [45].

### Behavioral analysis

For hindlimb suspension test, pups were placed face down into the standard 50 mL tube with their hind legs hung over the rim, observed the hindlimb posture, scored posture according to the criteria (4, normal hindlimb separation with tail raised; 3, apparent weakness and hindlimbs close together without touching each other; 2, closer hindlimbs, almost touching; 1, hindlimbs always clasping with the tail raised; 0, constant clasping of the hindlimbs with the tail lowered), and recorded the latency to fall and the number of pulls [46]. For negative geotaxis test, pups were placed with their head pointing downward on a 35° incline and hold it for 5 s, released and recorded the time and direction the pup turns to face upward [47]. For the tail suspension test (self-clasping test), pups were suspended by pulling their tails, observed the hindlimb posture, scored posture according to the criteria (4, normal, hindlimbs spread open; 3, hindlimbs not completely spread; 2, hindlimbs often close together; 1, hindlimbs always close together; 0, clasping, hindlimbs always close together with postural abnormalities), and recorded the clasping time [48]. For righting reflex test, pups were placed on their backs on a bench pad, held in position for 5 s, released and recorded the time it takes the pups to return to prone position as well as the direction righting [47].

### Histology

Mouse tissues were fixed with 4% paraformaldehyde, embedded in paraffin, and sectioned at 5 µm. Sections were stained with H&E stain, Nissl stain or subjected to immunohistochemical stain as described previously [18]. For immunohistochemical staining of spinal cord and brain sections, tissues were permeabilized in 0.25% Triton-X in TBS at room temperature for 15 min. Antigen retrieval was performed using microwave method in 10 mM citrate buffer (pH 6.0). Sections were blocked in blocking buffer (2% normal goat serum in TBST) at room temperature for 60 min and then incubated with primary antibodies at 4 °C, overnight. The following primary antibodies were used: anti-ChAT antibody (Abcam, #ab34419, 1:50 and #ab178850, 1:2000), anti-NeuN antibody (Abcam, #ab177487, 1:300), anti-GFAP antibody (Cell Signaling Technologies, #3670S, 1:800), anti-MAG antibody (Cell Signaling Technologies, 1:500), anti-Iba1 antibody (Wako, 013-27691, 1:500), anti-cleaved caspase 3 antibody (Cell Signaling Technologies, #9661S, 1:400) and anti-LC3B antibody (Abcam, #ab48394, 1:400)). The secondary antibodies used were: Alexa Fluor 546 anti-Rabbit IgG (Invitrogen, #A11035, 1:400), Alexa Fluor 488 anti-Rabbit IgG (Invitrogen, #A11008, 1:400), Alexa Fluor 546 anti-Mouse IgG (Invitrogen, #A11003, 1:1000), Alexa Fluor 546 anti-Chicken IgY (Invitrogen, #A11040, 1:1000) and Alexa Fluor 488 anti-Chicken IgY (Invitrogen, #A11039, 1:1000) were used. Tissues were counter-stained with 4′,6-Diamidino-2-phenylindole, dihydrochloride (DAPI; DOJINDO, 340-07971, 1:1000) and was incubated for 120 min at room temperature. Immunofluorescence images were captured using fluorescence microscope (Keyence, BZ-X810).

For NMJ staining, after perfusing and post-fixing with 4% paraformaldehyde, whole muscles were dissected and teased into layers five to 10 fibers thick [49]. NMJs were immunolabeled with anti-neurofilament antibody (Sigma-Aldrich, #AB5539, 1:1000) for nerves, anti-synaptophysin antibody (Abcam, #ab14692, 1:150) for presynaptic terminals, Alexa Fluor 594-conjugated BTX (Invitrogen, #B13423, 1:200) for AChRs, Alexa Fluor 488 anti-Rabbit IgG (Invitrogen, #A11008, 1:1000) and Alexa Fluor 488 anti-Chicken IgG (Invitrogen, #A11039, 1:1000). The proportion of innervation patterns; “fully innervated”, “partially innervated” or “denervated” was quantified according to the staining patterns of AChRs and nerve terminals [50]. Immunofluorescence images were acquired with confocal microscope (Carl Zeiss, LSM710).

### Bioinformatics

We downloaded raw fastq files from the Gene Expression Omnibus database (GSE112771, GSE209926, PRJNA1076644, GSE76220 and SRP064478) and the BioStudies database (E-MTAB-3664) [25–28, 51, 52]. First, we performed the quality check and trimmed low-quality reads using falco (version 1.2.3) and fastp (version 0.23.3), respectively. Then, STAR aligner (version 2.7.10b) was used to map the reads to the reference genome (GRCm39 (mouse) or GRCh38 (human)), and read count was performed using RSEM (version 1.3.1). We identified the DEGs using the exact test implemented in the edgeR package [53]. GO analysis and GSEA were performed using the clusterProfiler package [54]. In this study, we used differentially expressed genes (false discovery rate (FDR) < 0.05 and |log_2_(fold change (FC))| > 0.5) for GO analysis. The EnhancedVolcano package was used to create volcano plots.

### Statistical analysis

Unless otherwise specified, presented as mean ± standard deviation (SD), and statistical significance was determined by the two-tailed Student’s *t* test or the one-way or two-way ANOVA with Wilcoxson’s rank sum test. Survival of mice was evaluated by Kaplan-Meier analysis.

## Supplementary information

Supplemental information
