# Supplementary material for: The amino acid transporter LAT1 coordinates proper motor function at the perinatal stage
Source: Cell Death Dis. 2026 Mar 24;17(1):345. doi: 10.1038/s41419-026-08663-8 (PMC13039311; doi:10.1038/s41419-026-08663-8)
Supplement: Supplementary file 1 — Supplemental information [file 41419_2026_8663_MOESM1_ESM.pdf]

Supplemental Figure 1

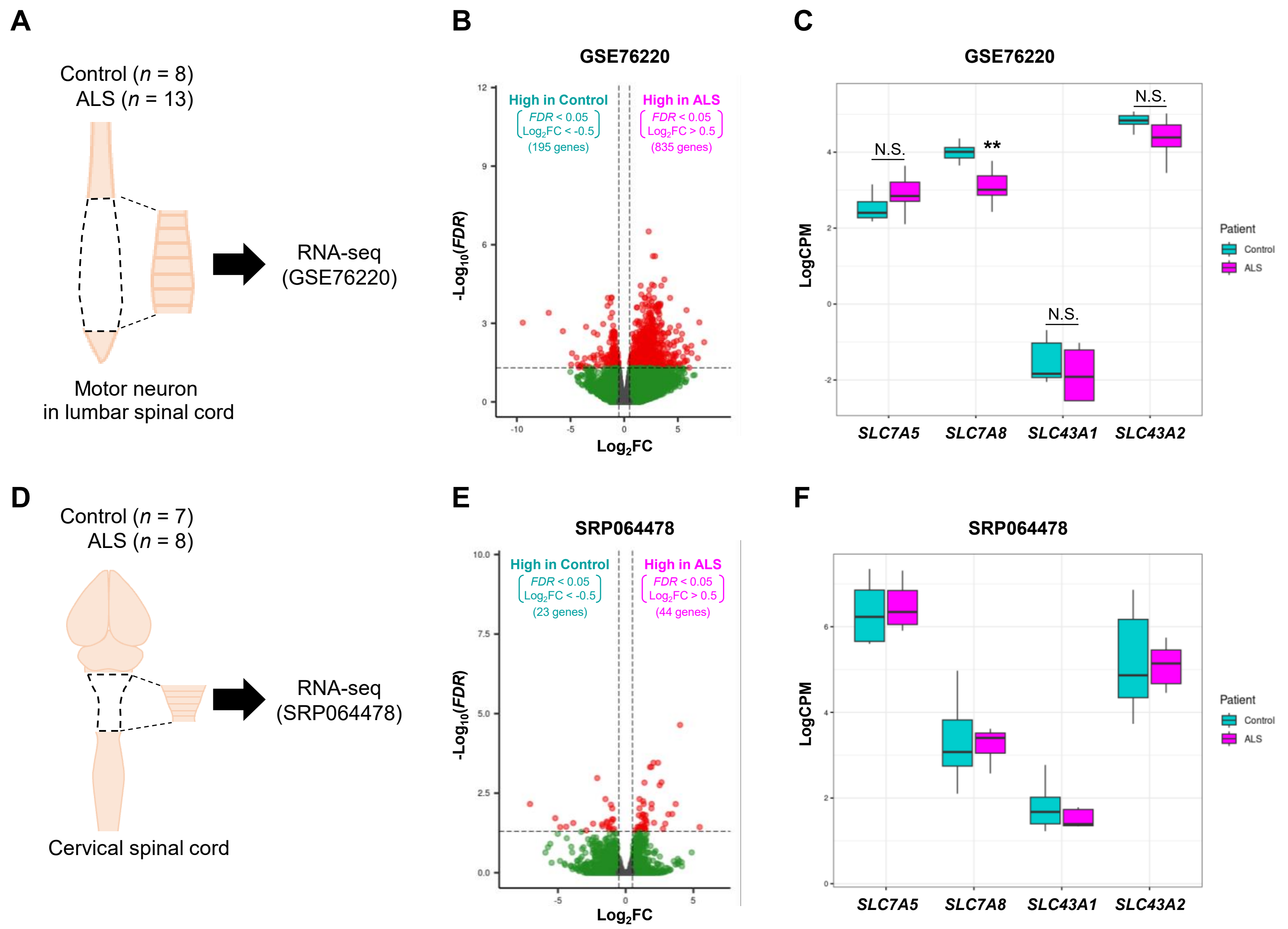

Supplemental Figure 1.
(A) Schematic diagram of sample information and RNA-seq analysis of the GSE76220 dataset. **(B and C)** Volcano plot and the expression of *SLC7A5* (LAT1), *SLC7A8* (LAT2), *SCL43A1* (LAT3), *SLC43A2* (LAT4) in the GSE76220 dataset (Control, *n* = 8; ALS, *n* = 13). **(D)** Schematic diagram of sample information and RNA-seq analysis of the SRP064478 dataset. **(E and F)** Volcano plot and the expression of *SLC7A5* (LAT1), *SLC7A8* (LAT2), *SLC43A1* (LAT3), *SLC43A2* (LAT4) in the SRP064478 dataset (Control, *n* = 7; ALS, *n* = 8).
